# Age- and Sex-Associated Variations in the Sensitivity of Serological Tests Among Individuals Infected With SARS-CoV-2

**DOI:** 10.1001/jamanetworkopen.2021.0337

**Published:** 2021-02-12

**Authors:** Rohit Vashisht, Ayan Patel, Bridgit O. Crews, Omai B. Garner, Lisa Dahm, Charles Wilson, Atul J. Butte

**Affiliations:** 1 Bakar Computational Health Sciences Institute, University of California, San Francisco; 2 Center for Data-driven Insights and Innovation, University of California Health, Oakland; 3 Department of Pathology and Laboratory Medicine, University of California, Irvine; 4 Department of Pathology and Laboratory Medicine, UCLA Medical Center, Los Angeles, California

## Abstract

This cohort study examines the sensitivity of antibody tests to detect previous severe acute respiratory syndrome coronavirus 2 (SARS-CoV-2) infection by time, test, sex, and age.

## Introduction

Antibodies against severe acute respiratory syndrome coronavirus 2 (SARS-CoV-2) are known to appear 2 to 3 weeks after infection,^1,2,3^ but patient characteristics and measurement timing could influence this immune response. This cohort study investigated the sensitivity of antibody tests to detect previous SARS-CoV-2 infection using existing clinical data across the University of California Health (UC Health) system.

## Methods

The institutional review boards across the UC Health system determined this cohort study was not human participants research and therefore was exempt from approval and informed consent. This study is reported following the Strengthening the Reporting of Observational Studies in Epidemiology (STROBE) reporting guideline.

The UC Health system treats the general population across 6 academic health centers and 12 hospitals, with approximately 150 000 inpatient and 4 million outpatient visits yearly. Data for this cohort study were drawn from the UC coronavirus disease 2019 (COVID-19) research data set (UC CORDS), a Health Insurance Portability and Accountability Act–limited data set comprising more than one-quarter–million patients tested for SARS-CoV-2 in any inpatient or outpatient setting. Three types of clinical immunoglobulin G (IgG) measurements were obtained between February 1 and October 15, 2020, in patients with real-time reverse transcription-polymerase chain reaction (RT-PCR) confirmation of SARS-CoV-2 infection. The sensitivity of the antibody test was calculated in 7-day increments from the positive RT-PCR test. We used *t* test to compare sensitivity by patient-reported sex. Analysis of variance was used to compare sensitivity by test types and age groups, followed by pairwise comparison using Tukey Honest Significant Differences. *P* values were 2-sided and corrected for multiple hypotheses. Statistical significance was set at *P* < .05. Analyses were conducted using R statistical software version 3.6.3 (R Project for Statistical Computing). Data were analyzed from August 1, 2020, to October 20, 2020.

## Results

Across the UC Health system, 277 567 patients were tested via RT-PCR for SARS-CoV-2 infection (mean [SD] age 47.0 [21.0] years; 150 133 [54.1%] women), and 14 290 were tested for SARS-CoV-2 IgG antibodies (Table). Of 10 065 patients with RT-PCR results positive for SARS-CoV-2 infection (mean [SD] age 41.4 [19.9] years; 5165 [51.3%] women), 486 patients (4.8%) underwent subsequent SARS-CoV-2 antibody testing a median (interquartile range) of 34 [3-64] days later (Figure, A). Of these, 365 patients (75.1%) had test results positive for antibodies. Antibody response significantly varied based on when the measurement was made. Patients whose serological tests were conducted closer to their positive RT-PCR results were more likely to have negative serological results than those tested later (Figure, A). The likelihood of positive SARS-CoV-2 antibody test results increased with time between the positive RT-PCR result and the antibody test, with sensitivity reaching 0.75 (95% CI, 0.71-0.79) at 112 days after the positive RT-PCR result (Figure, B)

**Table.** Study Population Tested for SARS-CoV-2 RNA by RT-PCR, Tested for Antibodies Against SARS-CoV-2, and Tested for Antibodies After Positive RT-PCR Result

Characteristic	Patients, No. (%)					
	Tested for SARS-CoV-2 RNA with RT-PCR		Tested for antibodies against SARS-CoV-2		With positive RT-PCR results then tested for antibodies	
	RT-PCR negative (n = 267 502)	RT-PCR positive (n = 10 065)	IgG negative (n = 13 129)	IgG positive (n = 1161)	IgG negative (n = 121)	IgG positive (n = 365)
Sex						
Female	144 968 (54.2)	5165 (51.3)	6970 (53.1)	605 (52.1)	70 (57.9)	184 (50.4)
Male	122 534 (45.8)	4900 (48.7)	6159 (46.9)	556 (47.9)	51 (42.1)	181 (49.6)
Age group, y						
0-18	27 676 (10.3)	1019 (10.1)	369 (2.81)	34 (2.93)	3 (2.48)	4 (1.10)
19-39	72 226 (27.0)	3865 (38.4)	3610 (27.5)	349 (30.1)	49 (40.5)	113 (31.0)
40-49	34 276 (12.8)	1606 (16.0)	2557 (19.5)	214 (18.4)	13 (10.7)	62 (17.0)
50-59	41 362 (15.5)	1547 (15.4)	2643 (20.1)	223 (19.2)	15 (12.4)	79 (21.6)
60-69	43 749 (16.4)	1032 (10.3)	2318 (17.7)	208 (17.9)	17 (14.0)	52 (14.2)
≥70	48 213 (18.0)	996 (9.90)	1632 (12.4)	133 (11.5)	24 (19.8)	55 (15.1)

Abbreviations: IgG, immunoglobin G; RT-PCR, reverse transcription–polymerase chain reaction; SARS-CoV-2, severe acute respiratory syndrome coronavirus 2.

**Figure.**
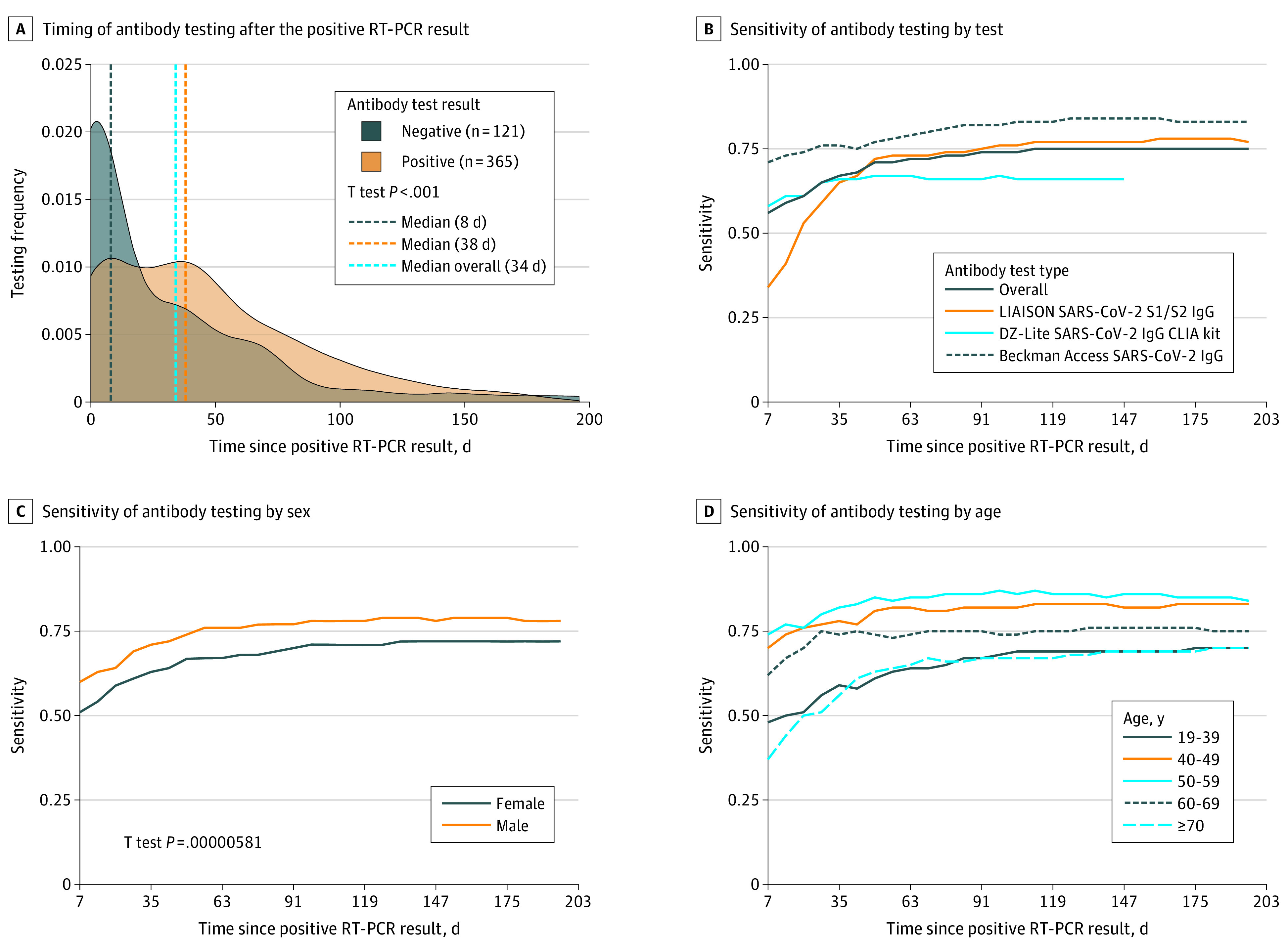
Timing and Sensitivity of Severe Acute Respiratory Syndrome Coronavirus 2 (SARS-CoV-2) Antibody Testing LIAISON SARS-CoV-2 S1/S2 IgG (Diasorin) tests were used February 1 to October 15, 2020; DZ-Lite SARS-CoV-2 IgG CLIA kit (Diazyme), February 1 to August 31, 2020; and Beckman Access SARS-CoV-2 IgG, September 1 to October 15, 2020 (B). RT-PCR indicates reverse transcription–polymerase chain reaction.

The sensitivity varied significantly by test type. The Beckman Coulter SARS-CoV-2 IgG test had a maximum sensitivity of 0.84 (95% CI, 0.73-0.91), the LIAISON SARS-CoV-2 S1/S2 IgG (Diasorin) test had a maximum sensitivity of 0.78 (95% CI, 0.72-0.82), and the DZ-Lite SARS-CoV-2 IgG CLIA kit (Diazyme) test had a maximum sensitivity of 0.67 (95% CI, 0.57-0.75) (Figure, B).

The sensitivity of the antibody test varied by sex and age, with significantly higher sensitivity among males (0.79; 95% CI, 0.73-0.84) than among females (0.72; 95% CI, 0.66-0.77; Cohen *d* = 1.32; 95% CI, 0.8-2.13; *P* < .001) (Figure, C). The sensitivity was highest at 126 days after positive RT-PCR results for males and 133 days after positive RT-PCR results for females. The sensitivity also varied significantly by age group, with the highest sensitivity among patients aged 50 to 59 years (0.87; 95% CI, 0.78-0.94) followed those aged 40 to 49 years (0.83; 95% CI, 0.72-0.91; Cohen *d* = 0.97; 95% CI, 0.41-1.72; *P* < .001) (Figure, D). Nearly all pairwise comparisons of antibody test sensitivities across age groups were significantly different (η^2^ = 0.71; 95% CI, 0.64-0.76; Tukey Honest Significant Difference, *P* < .001) except between the youngest and oldest groups and between patients aged 40 to 49 years vs 50 to 59 years.

## Discussion

The findings of this cohort study suggest that measuring serological levels too soon after SARS-CoV-2 infection might lead to an incorrect assessment of immune response. The sensitivity of antibody testing was higher in males and patients aged 40 to 59 years, but our subset cohort sizes are small. If these findings are validated, then accounting for differences in sex and age in interpreting serological levels may be warranted. Antibody test sensitivity was stable at 112 days and beyond after a known positive RT-PCR result, suggesting a detectable antibody response long after infection.^4,5^

This study has some limitations. The study was performed in a limited-size convenience cohort of patients. Preexisting conditions, clinical severity of disease, and unobserved clinical factors may confound estimated sensitivity. Treatment centers were all in California, which may limit generalizability of the findings. Additionally, serological results were treated as binary observations; specific titer levels were not available. The results of this cohort study suggest that while it was possible to conduct serological tests too soon after SARS-CoV-2 infection, a detectable antibody response was clinically detectable many months after an infection.
